# Detection of imprinting effects for hypertension based on general pedigrees utilizing all affected and unaffected individuals

**DOI:** 10.1186/1753-6561-8-S1-S52

**Published:** 2014-06-17

**Authors:** Fangyuan Zhang, Shili Lin

**Affiliations:** 1 Department of Statistics, The Ohio State University, 1958 Neil Avenue Columbus, OH, 43210, USA

## Abstract

Imprinting effects can lead to parent-of-origin patterns in many complex human diseases. For hypertension, previous studies revealed the possible involvement of imprinted genes. Genetic Analysis Workshop 18 real data, with hypertensive phenotype and genotype of more than 1000 individuals from 20 pedigrees, provided us an opportunity to further substantiate such findings. To test for imprinting effects, we developed a pedigree-parental-asymmetry test taking both affected and unaffected offspring into consideration (PPATu). We carried out a simulation study based on the Genetic Analysis Workshop 18 pedigrees to show that PPATu has well-controlled type I error and is indeed more powerful than the pedigree-parental-asymmetry test (PPAT), an existing method that does not utilize information from unaffected offspring. We then applied PPATu to Genetic Analysis Workshop 18 genome-wide association study data from 20 pedigrees. We identified a number of single-nucleotide polymorphisms showing significant imprinting effects that are within genomic regions that have been previously implicated to be associated with hypertension.

## Background

Genomic imprinting refers to the phenomenon of unequal expression of a heterozygous genotype depending on which parent (father or mother) the imprinted variant is inherited from. It is estimated that approximately 1% of all mammalian genes are imprinted (http://igc.otago.ac.nz). Among these imprinted genes, Beckwith-Wiedemann syndrome, Silver-Russell syndrome, Angelman syndrome, and Prader-Willi syndrome are the best known.

Numerous methods have been proposed to detect imprinting effects. For a diallelic genetic marker locus, the parental-asymmetry test (PAT) that considers imbalance of parental origins of the variant allele is simple and powerful. A series of generalizations of PAT, such as the pedigree-parental-asymmetry test (PPAT) for general pedigree data, widen its practical range [1]. However, these tests use only information on affected offspring and their parents. Recently, PATu [2] was proposed to take unaffected offspring in a nuclear family into consideration, making fuller use of data to improve power. In this study, we propose a novel parent-of-origin effects test, PPATu, that uses both affected and unaffected offspring in general pedigrees, and apply the method to the Genetic Analysis Workshop 18 (GAW18) data, consisting of 20 large pedigrees, to study the hypertensive phenotype. Previous studies revealed the possible involvement of imprinted genes in hypertension [3,4]. The GAW18 data thus provide us the opportunity to further substantiate such findings.

## Methods

Suppose that the marker of interest has 2 alleles, M_1 _and M_2_, and the disease allele is more likely to be associated with marker allele M_1_. Let 0, 1, and 2 represent the marker genotypes M_2_M_2_, M_1_M_2_, and M_1_M_1_, respectively. For a child-parents trio, let F, M, and C denote the marker genotypes of the father, mother, and child, respectively. Throughout this article, mating symmetry is assumed; that is, P(F=f, M=m)=P(F=m, M=f) for all f, m = 0,1,2. We also assume that there is no maternal effect; that is, the maternal genotype does not confer additional risk on the child's phenotype.

Suppose we have *N *independent pedigrees, and for the *i*^th ^pedigree, we have *nu_i _*unaffected and *na_i _*affected offspring. Define $S=\overset{N}{\underset{i=1}{\sum }}\left[\overset{na_{i}}{\underset{j=1}{\sum }}\left({I_{F_{ij}}}_{>M_{ij},C_{ij=1}}-I_{F_{ij}<M_{ij},C_{ij}=1}\right)-\overset{nu_{i}}{\underset{k=1}{\sum }}\left(I_{F_{ik}>M_{ik=1},C_{ik=1}}-I_{F_{ik}<M_{ik},C_{ik=1}}\right)\right]$

where *I *is the usual indicator function. We can prove that under the null hypothesis of no imprinting effect, *E*(*S*) = 0. The unbiased estimator of the variance of *S *is

$$\hat{V}\left(S\right)=\overset{N}{\underset{i=1}{\sum }}{\left[\overset{na_{i}}{\underset{j=1}{\sum }}\left({I_{F_{ij}}}_{>M_{ij},C_{ij=1}}-I_{F_{ij}<M_{ij},C_{ij}=1}\right)-\overset{nu_{i}}{\underset{k=1}{\sum }}\left(I_{F_{ik}>M_{ik},C_{ik=1}}-I_{F_{ik}<M_{ik},C_{ik=1}}\right)\right]}^{2}$$

The standardized test statistic $PPATu=\frac{S}{\sqrt{\hat{V}\left(S\right)}}$ follows the N(0, 1) distribution asymptotically. When there is maternal imprinting effect, PPATu will be positive; when there is paternal imprinting effect, it will be negative. Note that the contributions from trios in a pedigree are not independent, and their correlations are accounted for in the variance. In our simulation study and application below, we compare the performance of PPATu with PPAT, whose statistic is defined without the negative terms in the *S *statistic; that is, without utilizing information on trios with unaffected offspring. More specifically, $PPAT=\frac{\overset{N}{\underset{i=1}{\sum }}\overset{na_{i}}{\underset{j=1}{\sum }}\left(I_{F_{ij}>M_{ij},C_{ij}=1}-I_{F_{ij}<M_{ij},C_{ij}=1}\right)}{\sqrt{\overset{N}{\underset{i=1}{\sum }}{\left[\overset{na_{i}}{\underset{j=1}{\sum }}\left(I_{F_{ij}>M_{ij},C_{ij}=1}-I_{F_{ij}<M_{ij},C_{ij}=1}\right)\right]}^{2}}}$

## Results

### Simulation study

**Figure 1:**
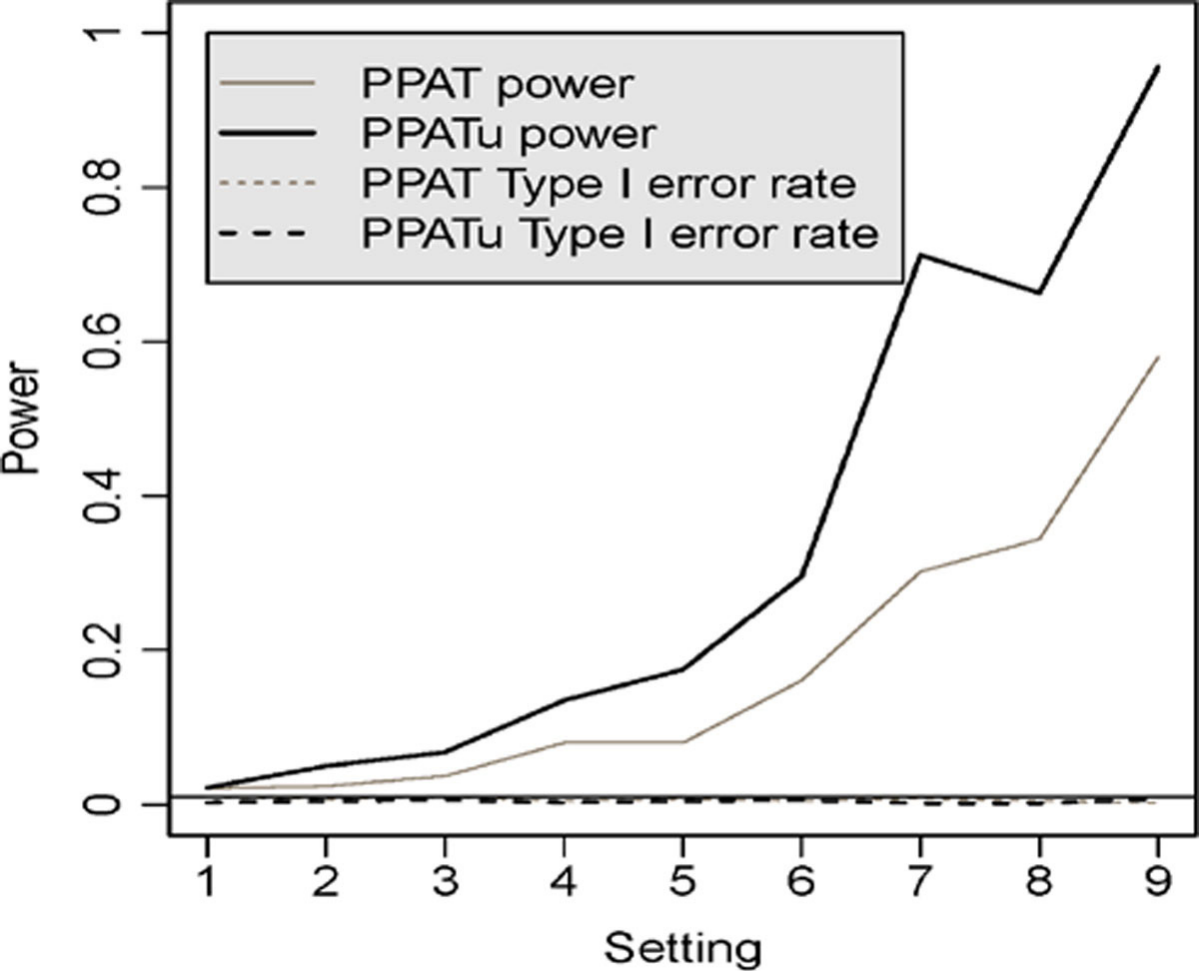
**Type I error and power for PPATu and PPAT**. The solid horizontal line marks the nominal significance level of 0.01. For power, the 9 settings are the 9 combinations of haplotype frequencies and imprinting models (as given in Table 1): 1 = (H1, I1), 2 = (H2, I1), 3 = (H3, I1), 4 = (H1, I2), 5 = (H2, I2), 6 = (H3, I2), 7 = (H1, I3), 8 = (H2, I3), and 9 = (H3, I3). For type I error, the 9 settings are the 9 combinations of haplotype frequencies and the no-imprinting models (as given in Table 1): 1 = (H1, N1), 2 = (H2, N1), 3 = (H3, N1), 4 = (H1, N2), 5 = (H2, N2), 6 = (H3, N2), 7 = (H1, N3), 8 = (H2, N3), and 9 = (H3, N3).

To evaluate the power of the proposed statistic and to compare with PPAT, we carried out a simulation study under 9 different settings, combinations of 3 sets of haplotype frequencies (H1, H2, H3) and 3 imprinting models (I1, I2, I3) (Table 1). Our data were simulated based on the general pedigrees from GAW18; their sizes are described below. To gauge the type I error rate, we also considered 9 additional settings, combinations of the same 3 sets of haplotype frequencies and 3 no-imprinting models (N1, N2, N3), also given in Table 1. We simulated 1000 replicates under each of the settings (a total of 18 combinations). The results are plotted in Figure 1, which shows that the empirical type I error rates, at the 0.01 nominal significance level, are all well controlled for both PPAT and PPATu. On the other hand, PPATu is clearly more powerful in all settings, especially when there is a substantial imprinting effect. Conclusions are the same for significance levels 0.05 and 0.005 as well; consequently,the results are not shown for brevity.

**Table 1:** Combinations of 9 imprinting settings and 9 no-imprinting settings

	Haplotype frequency^a^					Imprinting model^b^					No-imprinting model^b^		
					
	DM1	dM1	DM2	dM2		$\varphi_{d/d}$	$\varphi_{d/D}$	$\varphi_{D/d}$	$\varphi_{D/D}$		$\varphi_{d/d}$	$\varphi_{d/D}$	$\varphi_{D/d}$
H1	0.2	0.0	0.1	0.7	I1	0.26	0.28	0.37	0.39	N1	0.26	0.33	0.39

H2	0.3	0.1	0.0	0.6	I2	0.24	0.26	0.42	0.44	N2	0.24	0.34	0.44

H3	0.3	0.0	0.0	0.7	I3	0.18	0.23	0.53	0.58	N3	0.18	0.38	0.58

^a^Haplotype frequencies are between disease susceptibility locus(with disease allele D and normal allele d) and marker locus (alleles M1 and M2).

^b^Imprinting models are defined in terms of penetrance probabilities: *(ϕ_d/d_, ϕ_d/D_, ϕ_D/d_, ϕ_D/D_*). For the no-imprinting models,*ϕ_D/d _= ϕ_d/D_*.

### GAW18 data analysis

We consider GAW18 real genome-wide association studies pedigree data that contain a total of 472,049 single-nucleotide polymorphism (SNP) genotypes on odd chromosomes and phenotype data, including systolic and diastolic blood pressure (SBP and DBP). In our study, we use a hypertensive binary phenotype; an individual is classified as affected if SBP > 140 mm Hg, or DBP > 90 mm Hg, or on antihypertensive medication at the first examination. There are 20 pedigrees; the sizes range from 27 to 107 individuals. In total, there are only 157 affected offspring, while there are 709 unaffected ones. Hence, based on the experience gained in our simulation, we expect a substantial gain in information for PPATu that makes use of information from both affected and unaffected individuals when compared to PPAT. To reduce the effect of multiple testing, we first used pedigree disequilibrium test (PDT [5]) to identify SNPs that are associated with hypertension at the 0.05 level, and then performed imprinting effect tests, focusing only on those SNPs. In our analysis, all trios with complete data within each pedigree were included in computing the test statistic. Furthermore, although many tests were performed, we did not attempt to correct for multiple testing given the small sample size (a total of only 20 pedigrees).

**Figure 2:**
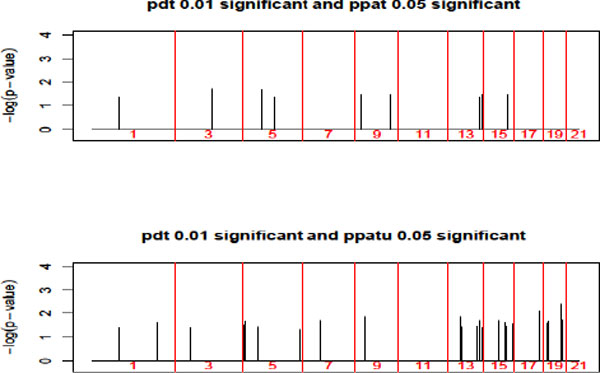
**Significant imprinting results (*p *value < 0.05) for SNPs having small *p *values for association test (815 SNPs with *p *value < 0.01) across all odd chromosomes**. (*Top*) Based on PPAT that uses only affected off spring and their parent data (17 SNPs identified). (*Bottom*) Based on PPATu that uses both affected and unaffected off spring (30 SNPs identified).

Table 2 shows the cross-classification of SNPs by different tests. We also provide Figure 2, which shows the results for a combination of significant levels. As one can see from Figure 2 and Table 2, PPATu detected more SNPs with smaller *p *values than PPAT. For SNPs detected by both methods, the results for PPATu are more significant. Table 3 shows the 2 SNPs (rs12947636 and rs1674137) that are significant at the 0.01 level for both PDT and PPATu. It appears that these 2 SNPs are novel, as they were not previously identified as associated with hypertension, thus further study is warranted to substantiate the finding.

**Table 2:** Cross-classification of results based on *p *values fromassociation(PDT) andimprinting (PPAT and PPATu) tests

	PPAT				PPATu		
PDT	<0.05	<0.01	>0.05		<0.05	<0.01	>0.05
<0.05	245	1	12,752		556	59	12,441
<0.01	17	0	798		30	2	785
>0.05	7008	35	14,681		15,007	1328	6682

**Table 3:** SNPs having *p *values <0.05 for both association and imprinting tests

SNP	Chr	Gene	Position	P__PDT_	P__PPAT_	P__PPATu_	Imprinting
rs12947636	17	*SLC39A11*	70992565	0.0090	0.6171	0.0080	maternal
rs1674137	19	*TSKS*	50258030	0.0089	0.6547	0.0038	maternal
rs11606492	11	*PLEKHA7*	17000241	0.0488	0.1797	0.0176	paternal

The first 2 SNPs are significant at the 0.01 level for PDT and PPATu. The third SNP is significant at the 0.05 level for PDT and PPATu, and is within the *PLEKHA7 *gene previously identified as associated with hypertension.

**Figure 3:**
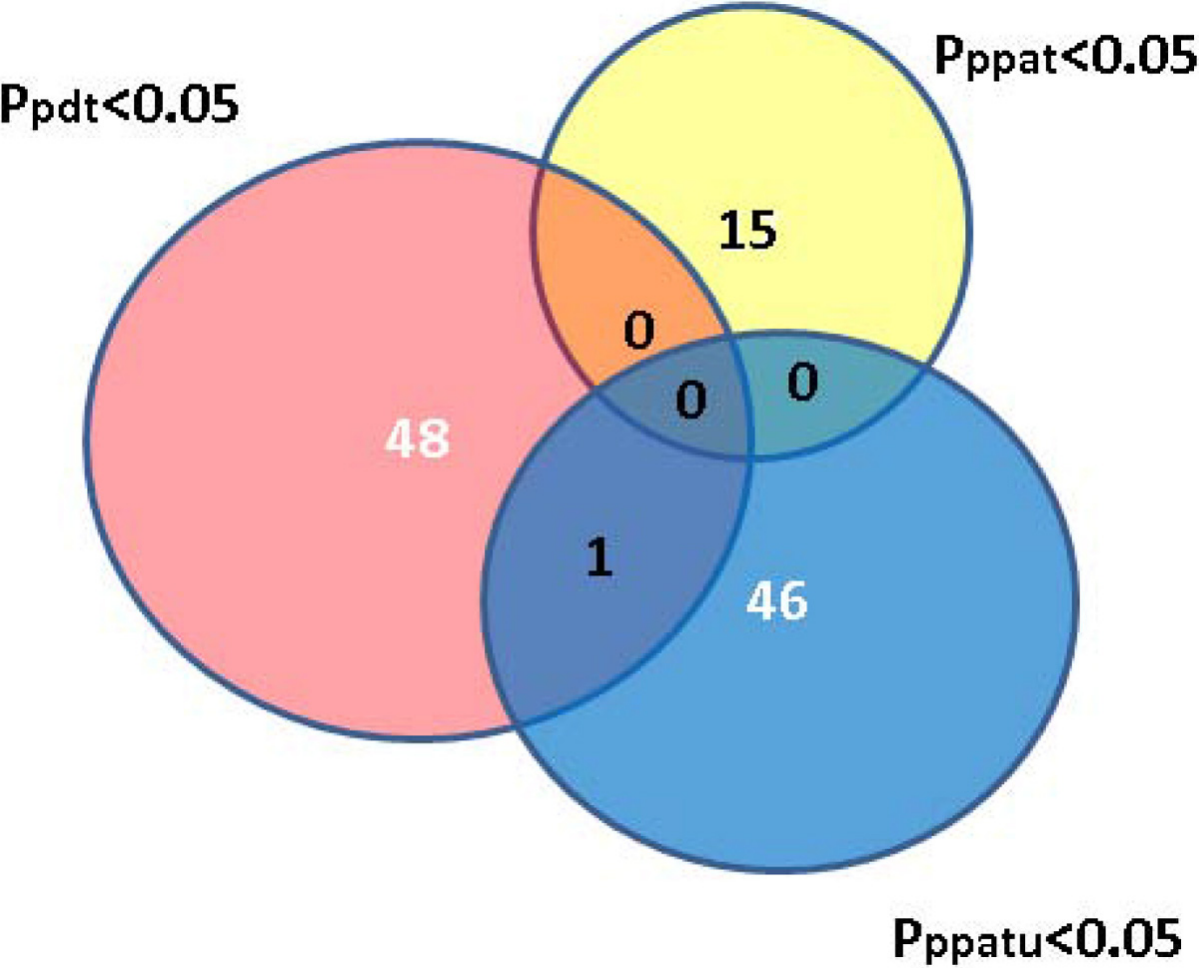
**Number of SNPs with significant imprinting or association effects (*p *value < 0.05) that are within genes previously identified as associated with hypertension**. The pink, yellow, and blue circles denote results from PDT, PPAT, and PPATu, respectively.

To gain a more global view of the extent of the role of imprinting in hypertension, we also carried out genome-wide testing for imprinting effects without restricting to SNPs with small *p *values from PDT. The analysis has resulted in larger sets with *p *values smaller than 0.05, which we call "significant"for easy reference. A search in the NCBI database found 49 genes implicated to be associated with hypertension in previous studies. Figure 3 gives the numbers of SNPs with significant imprinting or association effects within these genes. We can see that more SNPs identified by PPATu are within these genes than those identified by PPAT. Specifically, 47 and 15 SNPs within these genes are identified as having significant imprinting effects by PPATu and PPAT, respectively. In fact, the proportion of SNPs identified by PPATu that are in known genes is higher (1.5 times) than that identified by PPAT, although this difference is not substantial. Other than 1 SNP, the rest did not reach the threshold of significance for PDT, which may be explained by the power loss of association tests like PDT that do not account for the imprinting effect properly. The 1 SNP (rs11606492; Table 3) that yielded significant results from both PDT and PPATu (but not PPAT) is within the gene *PLEKHA7 *that has been implicated to be associated with hypertension in previous studies [6,7].

## Conclusions

In this article, we propose an imprinting test that utilizes both affected and unaffected individuals from general pedigrees, the type of data provided by GAW18. We expect PPATu to be more powerful than the existing test PPAT [1] because the former makes full use of information by taking unaffected offspring into consideration. Indeed, our simulation study shows that PPATu has higher power than PPAT without an elevated type I error rate based on the GAW18 pedigrees. Our results from analysis of the GAW18 data using PPATu leads to the identification of a number of SNPs that are within genomic regions previously implicated for the hypertensive phenotype. Nevertheless, further investigation is warranted especially to evaluate the performance of the methods under different study designs and ascertainment criteria.

## Competing interests

The authors declare that they have no competing interests.

## Authors' contributions

LS designed the overall study; FZ conducted statistical analyses and drafted the manuscript. Both authors wrote, read, and approved the final manuscript.
